# A Case Report of a Patient Surviving Severe Acidemia With Arterial pH Less Than 6.5

**DOI:** 10.7759/cureus.89135

**Published:** 2025-07-31

**Authors:** Karthik V Iyer, Justin A Jebackumar, Sarah Sundet, Moye Mathew, Vikram Oke

**Affiliations:** 1 Internal Medicine/Critical Care Medicine, Mercy Hospital Jefferson, Festus, USA; 2 Internal Medicine, Lake Erie College of Osteopathic Medicine, Erie, USA; 3 Internal Medicine, Mercy Hospital Jefferson, Festus, USA; 4 Pulmonary and Critical Care, Mercy Medical Center, Festus, USA

**Keywords:** ethylene glycol poisoning, fomepizole, hemodialysis, metabolic acidosis, severe acidemia

## Abstract

Surviving an arterial pH below 6.5 is exceedingly rare due to significant disruption of processes at the cellular level. We report a case of a 25-year-old female with altered mental status, hypotension, and refractory metabolic acidosis. Initial arterial blood gases were unreadable, but aggressive sodium bicarbonate therapy revealed an arterial pH of 6.62. Suspected ethylene glycol poisoning led to aggressive bicarbonate replacement, fomepizole therapy, and emergent hemodialysis. The patient showed rapid clinical recovery and was discharged from the hospital after six days, demonstrating that recovery is possible with appropriate, timely care even at potentially fatal arterial pH levels.

## Introduction

Metabolic acidosis is a commonly seen acid-base abnormality, with etiologies ranging from increased acid production, impaired acid excretion due to renal failure, bicarbonate loss, to ingestion of toxic substances [1,2]. Identifying the etiology of metabolic acidosis includes a thorough review of clinical manifestations and laboratory assessment. A prominent cause of anion gap metabolic acidosis (AGMA) is ingesting toxic substances such as salicylates, methanol, or ethylene glycol. Ethylene glycol is a clear, odorless, sweet-tasting liquid found in antifreeze and other household products. Even small ingestions (as little as 1.4 mL/kg) can be lethal, with a lethal time of 96 hours without treatment and a mortality rate approaching 20% [3,4]. In patients with glycolate ≤12 mmol/L or anion gap ≤28 mmol/L, mortality was 3.6%, highlighting the strong prognostic value of these markers [4]. Poisoning presents in three stages: central nervous system depression, cardiopulmonary complications, and renal failure [5]. Early symptoms can mimic ethanol or methanol intoxication but can progress rapidly if untreated. Diagnosis is based on high anion and osmolal gap, elevation in ethylene glycol blood level, and oxalate crystals in urine. Management includes antidotes (ethanol or fomepizole), correction of metabolic acidosis, and often hemodialysis in severe cases. Swift recognition and treatment are critical to reduce morbidity and mortality.

## Case presentation

A minimally responsive 25-year-old female was dropped off at the Emergency Department (ED) entrance of a local community hospital. She was unaccompanied and unable to provide history. Her initial Glasgow Coma Scale (GCS) score was 9, which quickly dropped to 5 [6]. The remainder of her physical examination, besides bibasilar rales on auscultation, was unremarkable. The patient presented with a blood pressure of 157/81 mmHg, respiratory rate of 18/min, heart rate of 87/min, temperature of 91.1°F, and oxygen saturation of 99% on room air. Initial laboratory findings (Tables 1, 2) suggested severe AGMA with an increased osmolal gap. Serum salicylate and acetaminophen levels were unremarkable (Table 3). Urine drug screen was positive for amphetamines and negative for other drugs (Table 4). Urinalysis demonstrated no ketones and no evidence of urinary tract infection.

**Table 1:** Chemistry AST, aspartate transaminase; ALT, alanine transaminase

Analyte	Patient Value	Reference Range	Units
Sodium	144	135–145	mmol/L
Potassium	5	3.5–5.1	mmol/L
Chloride	107	98–107	mmol/L
Bicarbonate	< 5	22–29	mmol/L
Blood glucose	222	70–99	mg/dL
Creatinine	1.84	0.6–1.1	mg/dL
Calcium	10.6	8.6–10.0	mg/dL
Albumin	5.2	4–5	mg/dL
AST	31	< 40	U/L
ALT	18	< 33	U/L
Lactic acid	10.6	< 2.0	mmol/L
Anion gap	>35	4–12	-
Osmolal gap	47	-10 to 10	mOsm/kg

**Table 2:** Hematology WBC, white blood cell; RBC, red blood cell

Analyte	Patient Value	Reference Range	Units
WBCs	74.1	4.0–11.0	K/μL
RBCs	5.94	4.2–5.4	M/μL
Hemoglobin	18.4	12.0–15.5	g/dL
Hematocrit	59.4	36–46	%
Platelets	538	150–450	K/μL

**Table 3:** Toxicology (blood)

Component	Patient Value	Reference Range	Units
Ethanol	<10.0	≤10	mg/dL
Acetaminophen	<5	10-30	ug/mL
Salicylate	<0.3	3-10	mg/dL

**Table 4:** Toxicology (urine)

Component	Patient Value	Reference Range
Amphetamine	Presumptive positive	Negative
Barbiturate	Negative	Negative
Benzodiazepine	Negative	Negative
Cocaine	Negative	Negative
Opioids	Negative	Negative
Phencyclidine	Negative	Negative
Oxycodone	Negative	Negative
Methadone	Negative	Negative

**Figure 1:**
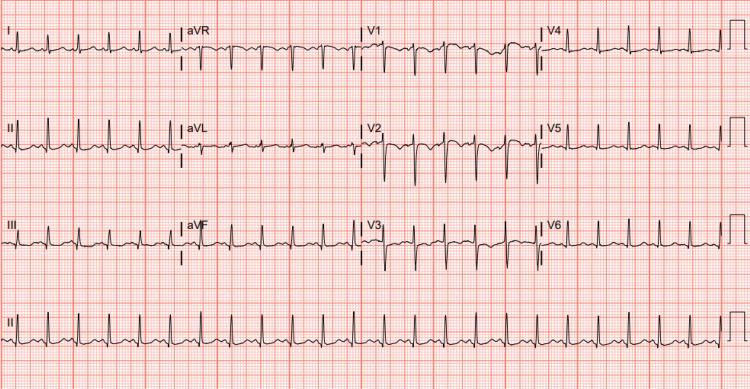
Electrocardiogram showing sinus tachycardia.

**Figure 2:**
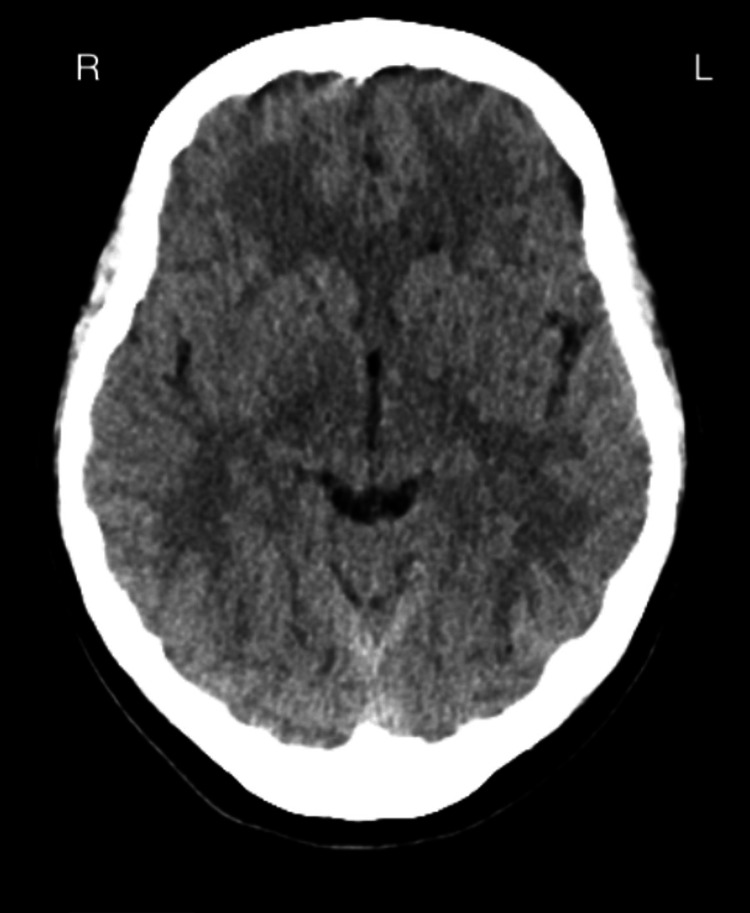
Non-contrast head CT showing no acute intracranial abnormalities. Diffusely hyperdense vascular structures are suggestive of dehydration.

**Figure 3:**
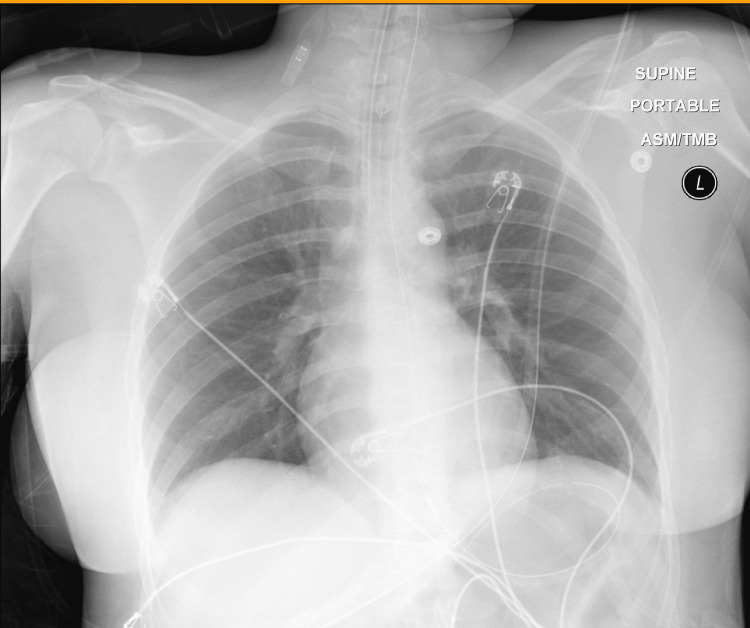
Chest X-ray showing no acute abnormalities.

Narcan was administered with no change in mental status. The patient was subsequently intubated for airway protection. Electrocardiogram revealed sinus tachycardia (Figure 1). Non-contrast head CT imaging revealed no acute intracranial abnormality (Figure 2). CT chest/abdomen/pelvis imaging without contrast did not reveal any acute abnormalities, and chest X-ray did not reveal any infiltrates (Figure 3). Multiple arterial blood gases (ABGs) drawn in the ED were uninterpretable, suggesting an extremely low pH (our ABG analyzers cannot detect pH < 6.5). She was admitted to the ICU and treated empirically for sepsis with broad-spectrum antibiotics and IV fluids. She developed refractory hypotension requiring norepinephrine, vasopressin, and phenylephrine infusion. Despite repeated bicarbonate administration, arterial pH continued to be undetectable. A critically low arterial pH of 6.62 was documented only after the administration of 400 mEq of sodium bicarbonate, approximately 3 hours following the initial uninterpretable ABG result (Table 5).

**Table 5:** Arterial blood gas

Component	Value	Normal Range	Units
pH arterial	6.62	7.35-7.45	-
PCO_2_ arterial	48	35-45	mm Hg
PO_2_ arterial	131	80-100	mm Hg
HCO_3_ arterial	5	22-26	mmol/L
Base excess	-33.3	-2.0-2.0	mmol/L

**Figure 4:**
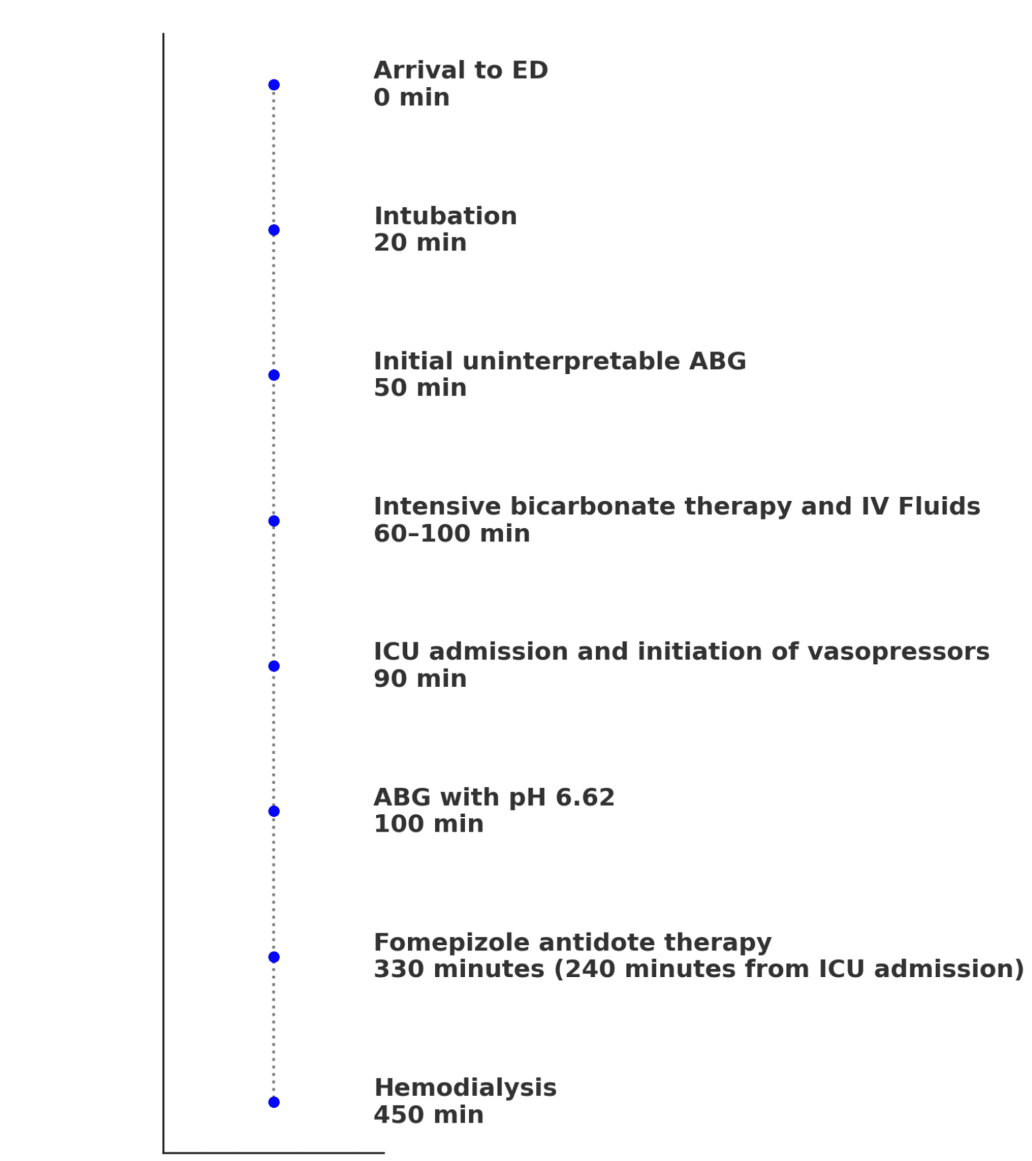
Clinical timeline of our patient

Given the refractory AGMA, elevated osmolal gap, and the absence of ketones or diabetic history, toxic ingestion was strongly suspected. ICU team suspected ethylene glycol poisoning based on presentation and laboratory findings. Fomepizole therapy was started at 15 mg/kg 4 hours after admission, and emergent hemodialysis was started 2 hours after antidote therapy initiation (Figure 4). Urinalysis results, unavailable until hemodialysis initiation, revealed urine calcium oxalate crystals. Serum ethylene glycol (drawn before hemodialysis) was later reported as 54.8 mg/dL (toxic level: >20.0 mg/dL), confirming the diagnosis. The patient’s family confirmed retrieving a voicemail with the patient's admission due to suicide attempt with antifreeze. With hemodialysis and fomepizole therapy, her severe acidosis resolved, serum osmolarity and ethylene glycol level normalized, and renal function improved. The patient was extubated on day 3 and discharged to behavioral health after six days, alert and oriented with no residual neurological deficits.

## Discussion

Arterial pH < 7.0 is associated with very high mortality, with rates as high as 57% to 90%, depending on etiology and intervention. Median time to death with arterial pH < 7 is approximately 13 hours [7]. Arterial pH under 6.8 is considered incompatible with life due to irreversible disruption of vital cellular processes [8,9]. Reports of full recovery at arterial pH < 6.5 are exceedingly rare [10]. Ethylene glycol toxicity results in CNS depression, metabolic acidosis, renal failure, and cardiopulmonary complications [3,11-12]. Survival at such extreme acidemia depends on rapid diagnosis, antidotal therapy, and emergent hemodialysis [7]. A thorough patient history remains the most effective tool for early identification of ingestion, particularly in unsupervised children and young adults with suicidal ideation [5]. Calcium oxalate monohydrate crystals, while not diagnostic on their own, can appear up to 4 days after ethylene glycol ingestion and, when present with high AGMA and suggestive history, strongly support the diagnosis [13]. Therapeutic interventions include fomepizole or ethanol, which competitively inhibit alcohol dehydrogenase, blocking toxic metabolite formation [11,14]. Fomepizole is now preferred for its predictable dosing and reduced side effects and is administered as a loading dose of 15 mg/kg followed by 10 mg/kg every 12 hours. [10,11,15]. Bicarbonate administration can help correct severe acidosis [2]. Hemodialysis is indicated for severe acidosis (arterial pH <7.0), acute kidney injury, or ethylene glycol levels >50 mg/dL [4]. Aggressive, early management with these interventions can prevent long-term complication and greatly improve the chances of survival.

## Conclusions

Profound metabolic acidosis can result from ingestion of toxins such as ethylene glycol. Survival at an arterial pH <6.5 is extremely rare but possible with prompt recognition and aggressive treatment. In this case, rapid diagnosis, administration of fomepizole, and hemodialysis resulted in a remarkable recovery. This case challenges the conventional belief that survival is impossible at such low pH and highlights the importance of considering toxic ingestion in patients with severe, unexplained metabolic acidosis. Clinicians must maintain a high index of suspicion and act swiftly to intervene in suspected poisoning cases to improve outcomes, even in settings of extreme physiological derangement.
